# Use of Cox's Cure Model to Establish Clinical Determinants of Long-Term Disease-Free Survival in Neoadjuvant-Chemotherapy-Treated Breast Cancer Patients without Pathologic Complete Response

**DOI:** 10.1155/2013/354579

**Published:** 2013-12-05

**Authors:** Junichi Asano, Akihiro Hirakawa, Chikuma Hamada, Kan Yonemori, Taizo Hirata, Chikako Shimizu, Kenji Tamura, Yasuhiro Fujiwara

**Affiliations:** ^1^Department of Management Science, Tokyo University of Science, Shinjuku-ku, Tokyo 162-8601, Japan; ^2^Center for Advanced Medicine and Clinical Research, Nagoya University Graduate School of Medicine, 65 Tsurumai-cho, Showa-ku, Nagoya 466-8560, Japan; ^3^Department of Breast and Medical Oncology, National Cancer Center Hospital, 5-1-1 Tsukiji, Chuo-ku, Tokyo 104-0045, Japan; ^4^Department of Respiratory Medicine, Okayama University Hospital, 2-5-1 Shikata-cho, Okayama 700-8558, Japan

## Abstract

In prognostic studies for breast cancer patients treated with neoadjuvant chemotherapy (NAC), the ordinary Cox proportional-hazards (PH) model has been often used to identify prognostic factors for disease-free survival (DFS). This model assumes that all patients eventually experience relapse or death. However, a subset of NAC-treated breast cancer patients never experience these events during long-term follow-up (>10 years) and may be considered clinically “cured.” Clinical factors associated with cure have not been studied adequately. Because the ordinary Cox PH model cannot be used to identify such clinical factors, we used the Cox PH cure model, a recently developed statistical method. This model includes both a logistic regression component for the cure rate and a Cox regression component for the hazard for uncured patients. The purpose of this study was to identify the clinical factors associated with cure and the variables associated with the time to recurrence or death in NAC-treated breast cancer patients without a pathologic complete response, by using the Cox PH cure model. We found that hormone receptor status, clinical response, human epidermal growth factor receptor 2 status, histological grade, and the number of lymph node metastases were associated with cure.

## 1. Introduction

Neoadjuvant chemotherapy (NAC) was introduced first in the early 1980s to improve tumor operability in patients with locally advanced breast cancers [1]. Recently, NAC applications have been extended to already-operable cases [1]. According to a previous meta-analysis of 9 randomized trials, NACs and adjuvant chemotherapies were equally effective in terms of overall survival (OS) and disease-free survival [2]. Breast cancer relapses or metastases were reported in only 34% of the patients within 8 years of NAC treatment [3], whereas a subset of NAC-treated primary breast cancer patients were reported to achieve long-term disease-free survivals (DFS) [3]. Accordingly, these patients did not experience recurrences, metastases, or death during the long-term follow-up study (e.g., for over 10 years) and were clinically “cured.” Although the prognostic factors for DFS have been established by several previous reports [4–9], clinical determinants of cure have not been studied adequately.

In prognostic studies for breast cancer patients treated with NAC, the Cox proportional-hazards (PH) model has been often used to identify the prognostic factors for DFS. The ordinary Cox PH model assumes that all patients will eventually experience relapse or death. Therefore, the ordinary Cox PH model cannot be used to identify the clinical factors associated with cure. To meet this requirement, we need to use the Cox PH cure model, a newly developed statistical method. The Cox PH cure model is well known in the field of statistics [10] but is not as widely known in the clinical setting [11]. This model includes both a logistic regression component for the cure rate and a Cox regression component for the hazard for uncured patients. Thus, the Cox PH cure model can potentially distinguish between clinical determinants of cure and variables associated with the time to recurrence or death. In this study, we used this model to identify the clinical determinants of cure (i.e., no relapse or death for at least 9 years) and the variables associated with the time to recurrence or death in patients without a pathologic complete response (pCR) who were treated with NAC.

## 2. Materials and Methods

### 2.1. Patients

We studied 459 primary breast cancer patients treated at the National Cancer Center Hospital, Tokyo, Japan, between May 1995 and July 2007. All patients had received anthracycline- or taxane-based NAC. The patients' clinical staging ranged from cT2N0 M0 to cT4dN3 M0, which included inflammatory (T4d) carcinomas. Data collected comprised pre- and post-NAC estrogen receptor (ER), progesterone receptor (PgR), and human epidermal growth factor receptor (HER) 2 expression levels in the tumors. Patients assessed for postoperative pCR (*N* = 91), including those with residual ductal carcinoma *in situ* (DCIS), were excluded because hormone-receptor (HR) status could not be accurately established in their tumors. Nine patients with unknown histological grade were excluded; the remaining patients (*N* = 359) were enrolled in the study. It is noteworthy that the same cohort had been previously studied by Hirata et al. [12] for evaluation of the impact of the change in the HR status on the long-term patient outcomes.

### 2.2. Determination of Hormone and HER2 Status

Patients underwent core needle biopsy (CNB) using an 18 G needle. ER, PgR, and HER2 status in the CNB or surgical specimens were ascertained by immunohistochemistry (IHC). Methodological details for IHC are summarized in Table 1. Positive staining for ER/PgR was defined as nuclear staining in ≥10% of the tumor cells. HER2 overexpression was defined as 3+ when complete membrane staining was detected. The Allred scoring system was used to assess the extent of staining [13]. If HER2 staining by IHC was found to be 2+ or less, fluorescent *in situ* hybridization (FISH) was used to confirm results. FISH was performed using the PathVysion kit (Abbott-Vysis Lab, Abbott Park, IL, USA). HER2 gene amplification was defined as a HER2: chromosome-17 ratio of 2.1. HR positivity was defined as positivity for ER and/or PgR. Standard IHC controls were prepared daily for each tumor staining.

### 2.3. Tumor Size Determination and Evaluation of Neoadjuvant Chemotherapy Response

Before each chemotherapy regimen and before surgery, the 2 greatest perpendicular diameters of the breast tumors and axillary lymph nodes were measured. The products of these diameters were added to give the total measure of tumor size. Absence of palpable breast tumors or axillary lymph nodes by clinical examination constituted a complete response (CR). Reduction in total tumor size by ≤50% constituted a partial response (PR). An increase in total tumor size by >50% or emergence of new, suspicious, ipsilateral axillary adenopathies indicated progressive disease (PD). Tumors that did not meet the criteria for objective responses or progression indicated stable disease (SD).

### 2.4. Chemotherapy

NAC comprised anthracycline- or taxane-based chemotherapy, either concurrently or sequentially. Patients receiving concurrent therapy were treated in 4 cycles (50 mg/m^2^ doxorubicin plus 60 mg/m^2^ docetaxel) every 21 days. Patients who achieved clinical CR or PR resulting from the above treatments underwent 2 additional cycles of the same treatment regimen postoperatively. However, patients with no objective clinical responses to NAC were treated with 4 cycles of 5-fluorouracil (600 mg/m^2^), methotrexate (40 mg/m^2^), and cyclophosphamide (600 mg/m^2^) postoperatively. Patients receiving the sequential regimen underwent 4 cycles of 5-fluorouracil (500 mg/m^2^), epirubicin (100 mg/m^2^), cyclophosphamide (500 mg/m^2^) or doxorubicin (60 mg/m^2^), and cyclophosphamide (600 mg/m^2^) every 21 days, followed by taxane-based chemotherapy. In the latter case, paclitaxel at 80 mg/m^2^ weekly for 12 weeks or at 175 mg/m^2^ every 3 weeks for 4 cycles or docetaxel every 3 weeks at 75 mg/m^2^ for 4 cycles was given.

### 2.5. Adjuvant Endocrine Therapy (ET) and Radiotherapy

Adjuvant radiotherapy was administered to patients who underwent breast-conserving surgery, that is, modified radical mastectomy for disease stages ranging from cT3N1 M0 to cT4dN3 M0. The decision to administer ET was taken by the responsible physician and/or based on the patients' preferences. Most patients with HR-positive lesions were given 20 mg tamoxifen daily for 5 years. From 2005 onwards, postmenopausal women taking tamoxifen were recommended to switch to an aromatase inhibitor before, after, or during the 5-year course of tamoxifen treatment.

### 2.6. Statistical Analysis

The demographic and clinical variables were documented for all the patients. DFS was defined as the time from surgery to date of disease relapse, death by any cause, or the date of the last clinical visit for uncomplicated patients. DFS rate was estimated using the Kaplan-Meier estimator. We used the Cox PH cure model to simultaneously estimate hazard ratios of the variables associated with the time to recurrence or death and odds ratios (ORs) of clinical criteria associated with cure (i.e., no relapse or death for at least 9 years) [10, 14]. According to the Cox PH cure model, ORs of >1 indicate a high proportion of patients who are cured, whereas hazard ratios of <1 indicate a relative improvement in disease-free survival among patients who are not cured. The 95% confidence interval (CI) for hazards ratios and ORs were also calculated. The stepwise variable selection method for the Cox PH cure model was used (*α* = 0.05) [15]. We applied the aforementioned analyses for 9-year disease-free patients. A 2-tailed *P* < 0.05 was considered to be statistically significant. We estimated parameters for the Cox PH cure model by using the EM algorithm [14] and uniquely programmed the SAS code accordingly using SAS/IML (version 9.3; SAS Institute Inc., Cary, NC, USA).

## 3. Results

### 3.1. Simultaneous Evaluations of Clinical Criteria Associated with Cure and the Variables Associated with the Time to Recurrence or Death

**Figure 1:**
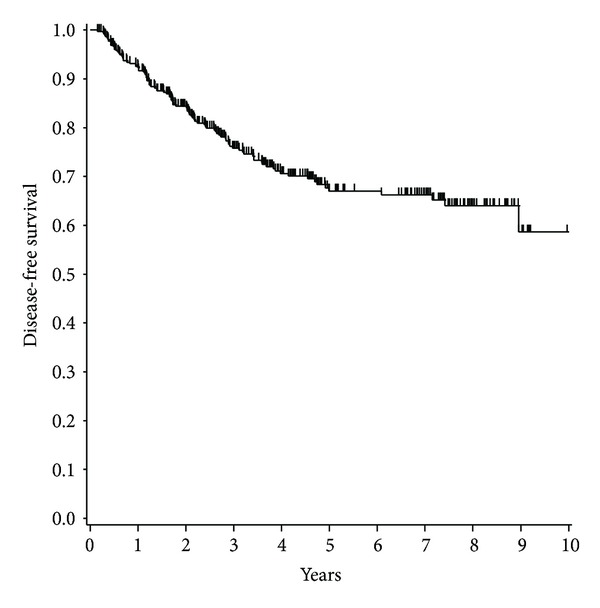
Kaplan-Meier analysis showing disease-free survival (*N* = 359). Short vertical lines indicate censored data points.

The patients' and tumor characteristics are listed in Table 2. The postoperative performance status (PS 0 or 1) of all the patients was good. None of the HER2-positive patients were treated with trastuzumab during neoadjuvant or adjuvant chemotherapy.  Figure 1 shows the Kaplan-Meier curves for DFS. The 5-, 7-, and 9-year DFS rates were 67.2%, 66.3%, and 58.7%, respectively.

**Figure 2:**
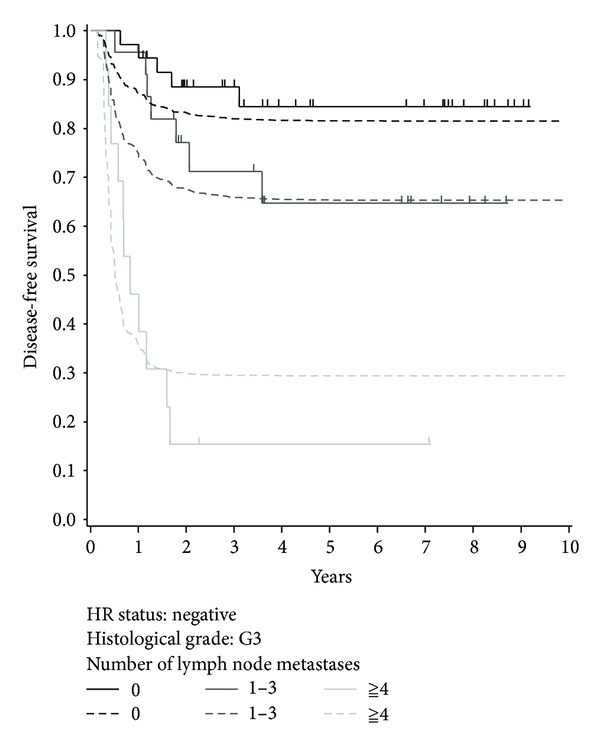
Kaplan-Meier curves (solid lines) and predicted survival curves calculated using the Cox PH cure model (dashed lines) for patients with histological grade 3 and negative HR status according to the number of lymph node metastases. Short vertical lines indicate censored data points.

Figure 2 shows the Kaplan-Meier curves (solid lines) for patients with histological grade 3 disease and negative HR status according to the number of lymph node metastases. The curves for the three groups were significantly different (*P* < 0.001, log-rank test).

Table 3 shows the results of the Cox PH cure model using stepwise variable selection. The following 8 variables were assessed: HR status (positive versus negative), ET (yes versus no), age (years), clinical stage at diagnosis (IIA, IIB, or IIIA versus IIIB or IIIC), histological grade (1 or 2 versus 3), HER2 status (positive versus negative), clinical response (CR versus PR versus SD or PD), and the number of lymph node metastases (0 versus 1–3 versus ≥4).

As shown in Table 3, five of these variables—HR status, clinical response, HER2 status, histological grade, and the number of lymph node metastases—were identified as factors associated with cure. All variables studied—HR status, ET therapy, age, clinical stage, clinical response, HER2 status, histological grade, and the number of lymph node metastases—were associated with the time to recurrence or death.

Table 4 shows the estimated proportion of cured patients, based on the Cox PH cure model, according to HR status, clinical response, HER2 status, histological grade, and the number of lymph node metastases. The estimated proportion of cured patients was more than 90% among those with a negative HR status, a CR, and no lymph node metastases.

Table 5 shows the results of analyses using the ordinary Cox PH model with the stepwise variable selection (*α* = 0.05). Six of these variables—HR status, ET therapy, clinical response, HER2 status, histological grade, and the number of lymph node metastases—were identified as factors associated with the time to recurrence or death.

## 4. Discussion

In this study, we identified variables that are associated with the time to recurrence or death and the clinical determinants of cure in NAC-treated patients without a pCR by using the Cox PH cure model. To our knowledge, this is the first study identifying the clinical criteria dictating cure in NAC-treated patients without pCR.

HR status, clinical response, HER2 status, histological grade, and the number of lymph node metastases were identified as the clinical criteria of cure and were found important in predicting clinical “cure.” The variables associated with the time to recurrence or death were HR status, ET, age, clinical stage at diagnosis, clinical response, HER2 status, histological grade, and the number of lymph node metastases. ET therapy and age were the only variables associated with recurrence or death. Clinical response was the variable associated with both the cure and the time to recurrence or death. However, the interval of the 95% CI of the hazard ratio for CR and that of the 95% CI of the OR for PR included 1. Thus, a CR was identified as a determining criterion for cure, whereas a PR was identified as a factor for determining the time to recurrence or death. By using the Cox PH cure model, we distinguished between variables associated with recurrence or death and the clinical determinants of cure in NAC-treated breast cancer patients.

We also found that more than 90% of patients with a negative HR status, a CR, and no lymph node metastases are expected to be cured (Table 4).

There are no established statistical methods for evaluating the goodness of fit of the Cox PH cure model. Here, we compared the Kaplan-Meier curves with the predicted survival curves based on the Cox PH cure model. Figure 2 also shows the predicted survival curves based on the Cox PH cure model. The Kaplan-Meier estimates of the DFS at 9 years for the groups with 0, 1–3, and ≥4 lymph node metastases were 84.5%, 64.8%, and 15.4%, respectively, and the corresponding cure rates estimated using the Cox PH cure model were 81.2%, 65.0%, and 29.4%, respectively.

Age and clinical stage were not identified as significant factors on using the ordinary Cox PH model, whereas those were identified as significant factors on using the Cox PH cure model. Thus, the Cox PH cure model could help identify variables associated with the time to recurrence or death that cannot be identified by using the ordinary Cox PH model.

## 5. Conclusion

We identified variables associated with the time to recurrence or death and the clinical factors associated with clinical outcome in NAC-treated patients without a pCR by using the Cox PH cure model. HR status, ET, age, clinical stage at diagnosis, clinical response, HER2 status, histological grade, and the number of lymph node metastases were identified as variables associated with the time to recurrence or death and HR status, clinical response, HER2 status, histological grade, and the number of lymph node metastases were identified as the clinical factors associated with cure. Age and clinical stage, not identified using the ordinary Cox PH model, were identified on using the Cox PH cure model. Additionally, more than 90% of patients with negative HR status, a CR, and no lymph node metastases are expected to be cured. Thus, the distinction between variables associated with the time to recurrence or death and the clinical factors associated with cure based on the Cox PH cure model can provide new insight with respect to the disease prognosis.

## Figures and Tables

**Table 1:** Experimental details used for immunohistochemistry.

Antigen	Period used for	Clone	Type	Antigen retrieval	Source
ER	Until Oct 2002	ID5	Mouse monoclonal	A/C citrate buffer	Dako
	From Nov 2002 to Feb 2005	ER88	Mouse monoclonal	As above	Bio Genex
	From Mar 2005	ID5	Mouse monoclonal	As above	Dako

PgR	Until Oct 2002	IA6	Mouse monoclonal	As above	Novocastra
	From Nov 2002 to Feb 2005	PR88	Mouse monoclonal	As above	Bio Genex
	From Mar 2005	PgR636	Mouse monoclonal	As above	Dako

HER2	Until Oct 2002	c-erbB-2	Rabbit polyclonal	As above	Dako
	From Nov 2002 to Feb 2005	CB11	Mouse monoclonal	As above	Bio Genex
	From Mar 2005	c-erbB-2	Rabbit polyclonal	As above	Dako

A/C: autoclave for 10 min at 121°C; ER: estrogen receptor; HER2: human epidermal receptor 2; PgR: progesterone receptor; citrate buffer: 10 mM citrate buffer, pH 6.0.

**Table 2:** Patient and tumor characteristics (*N* = 359).

Characteristics	
Age, years	
Mean ± SD	49.1 ± 9.4
Tumor stage	
T1	3 (0.8)
T2	173 (48.2)
T3	116 (32.3)
T4	67 (18.7)
*N* stage	
*N*0	176 (49.0)
*N*1	138 (38.4)
*N*2	35 (9.8)
*N*3	10 (2.8)
Clinical stage	
IIA	99 (27.6)
IIB	106 (29.5)
IIIA	80 (22.3)
IIIB	65 (18.1)
IIIC	9 (2.5)
Histological grade	
G1	20 (5.6)
G2	181 (50.4)
G3	158 (44.0)
Hormone receptor status	
Negative	120 (33.4)
Positive	239 (66.6)
PgR status	
Negative	200 (55.7)
Positive	159 (44.3)
ER status	
Negative	185 (51.5)
Positive	174 (48.5)
HER2 status	
Negative	237 (66.0)
Positive	122 (34.0)
NAC regimen	
AT	163 (45.4)
AC followed by T	86 (24.0)
CEF followed by T	110 (30.6)
Clinical response	
CR	90 (25.1)
PR	215 (59.9)
SD/PD	50 (13.9)
Operation	
Lumpectomy	123 (34.3)
Mastectomy	236 (65.7)
Radiotherapy	
Yes	239 (66.6)
No	120 (33.4)
Number of lymph node metastases	
0	145 (40.4)
1–3	114 (31.8)
≥4	100 (27.8)
Endocrine therapy	
TAM, 5 years	140 (39.0)
TAM followed by AI	54 (15.0)
AI, 5 years	33 (9.2)
None	132 (36.8)
ET therapy	
No	132 (36.8)
Yes	227 (63.2)

AC: doxorubicin and cyclophosphamide; AI: aromatase inhibitor; AT: doxorubicin and docetaxel; CEF: cyclophosphamide, epirubicin, and 5-fluorouracil; ER: estrogen receptor; HER2: human epidermal growth factor receptor 2; *N*: number of patients; PgR: progesterone receptor; T: taxane (weekly or triweekly paclitaxel or triweekly docetaxel); TAM: tamoxifen. Numbers in parentheses are percentage of patients except for age, which designates mean.

**Table 3:** Results of analyses using the Cox PH cure model with stepwise variable selection (*α* = 0.05).

Variables	Odds ratio (95% CI)	*P* value	Hazard ratio (95% CI)	*P* value
HR status		<0.001		0.019
Negative	1		1	
Positive	0.30 (0.17, 0.52)		2.83 (1.19, 6.73)	
ET therapy		Not selected		<0.001
No			1	
Yes			0.05 (0.02, 0.13)	
Age		Not selected	0.97 (0.95, 0.99)	0.013
Clinical stage		Not selected		0.007
IIA/IIB/IIIA			1	
IIIB/IIIC			1.90 (1.19, 3.03)	
Clinical response		0.003		0.004
SD/PD	1		1	
PR	0.82 (0.41, 1.65)		0.44 (0.26, 0.74)	
CR	2.25 (0.99, 5.07)		0.60 (0.29, 1.25)	
HER2 status		0.023		0.019
Negative	1		1	
Positive	0.54 (0.31, 0.92)		1.69 (1.09, 2.61)	
Histological grade		0.044		0.042
1 and 2	1		1	
3	0.59 (0.35, 0.98)		1.58 (1.02, 2.45)	
Number of lymph node metastases		<0.001		0.002
0	1		1	
1–3	0.43 (0.25, 0.74)		1.15 (0.61, 2.13)	
≥4	0.10 (0.05, 0.19)		2.21 (1.25, 3.90)	

**Table 4:** The estimated proportion of cured patients.

HR status	Clinical response	HER2 status	Histological grade	Number of lymph node metastases	Estimated proportion of cured patients
Negative	SD/PD	Negative	1 and 2	0	89.3%
				1 to 3	78.2%
				≥4	44.7%
			3	0	83.1%
				1 to 3	67.8%
				≥4	32.2%
		Positive	1 and 2	0	81.7%
				1 to 3	65.8%
				≥4	30.2%
			3	0	72.4%
				1 to 3	53.0%
				≥4	20.2%
	PR	Negative	1 and 2	0	87.3%
				1 to 3	74.6%
				≥4	39.8%
			3	0	80.1%
				1 to 3	63.4%
				≥4	28.0%
		Positive	1 and 2	0	78.6%
				1 to 3	61.2%
				≥4	26.2%
			3	0	68.3%
				1 to 3	48.1%
				≥4	17.2%
	CR	Negative	1 and 2	0	94.9%
				1 to 3	89.0%
				≥4	64.4%
			3	0	91.7%
				1 to 3	82.6%
				≥4	51.6%
		Positive	1 and 2	0	90.9%
				1 to 3	81.2%
				≥4	49.2%
			3	0	85.5%
				1 to 3	71.7%
				≥4	36.3%
Positive	SD/PD	Negative	1 and 2	0	71.2%
				1 to 3	51.5%
				≥4	19.3%
			3	0	59.2%
				1 to 3	38.4%
				≥4	12.3%
		Positive	1 and 2	0	57.0%
				1 to 3	36.3%
				≥4	11.3%
			3	0	43.8%
				1 to 3	25.1%
				≥4	7.0%
	PR	Negative	1 and 2	0	67.0%
				1 to 3	46.6%
				≥4	16.4%
			3	0	54.4%
				1 to 3	33.9%
				≥4	10.3%
		Positive	1 and 2	0	52.1%
				1 to 3	31.8%
				≥4	9.5%
			3	0	39.0%
				1 to 3	21.5%
				≥4	5.8%
	CR	Negative	1 and 2	0	84.7%
				1 to 3	70.5%
				≥4	34.9%
			3	0	76.5%
				1 to 3	58.4%
				≥4	24.0%
		Positive	1 and 2	0	74.8%
				1 to 3	56.1%
				≥4	22.3%
			3	0	63.6%
				1 to 3	42.9%
				≥4	14.4%

**Table 5:** Results of analyses using the ordinary Cox PH model with stepwise variable selection (*α* = 0.05).

Variables	Hazard ratio (95% CI)	*P* value
HR status		<0.001
Negative	1	
Positive	4.56 (1.92, 10.80)	
ET therapy		<0.001
No	1	
Yes	0.16 (0.07, 0.36)	
Age		Not selected
Clinical stage		Not selected
IIA/IIB/IIIA		
IIIB/IIIC		
Clinical response		0.026
SD/PD	1	
PR	0.60 (0.36, 0.99)	
CR	0.39 (0.19, 0.79)	
HER2 status		0.002
Negative	1	
Positive	1.97 (1.28, 3.03)	
Histological grade		0.002
1 and 2	1	
3	1.90 (1.26, 2.87)	
Number of lymph node metastases		<0.001
0	1	
1 to 3	1.93 (1.04, 3.58)	
≥4	7.33 (4.18, 12.85)	

## References

[1] Kaufmann M, Von Minckwitz G, Smith R (2003). International expert panel on the use of primary (preoperative) systemic treatment of operable breast cancer: review and recommendations. *Journal of Clinical Oncology*.

[2] Mauri D, Pavlidis N, Ioannidis JPA (2005). Neoadjuvant versus adjuvant systemic treatment in breast cancer: a meta-analysis. *Journal of the National Cancer Institute*.

[3] Rastogi P, Anderson SJ, Bear HD (2008). Preoperative chemotherapy: updates of national surgical adjuvant breast and bowel project protocols B-18 and B-27. *Journal of Clinical Oncology*.

[4] Brain E, Garrino C, Misset J-L (1997). Long-term prognostic and predictive factors in 107 stage II/III breast cancer patients treated with anthracycline-based neoadjuvant chemotherapy. *British Journal of Cancer*.

[5] Estévez LG, Gradishar WJ (2004). Evidence-based use of neoadjuvant taxane in operable and inoperable breast cancer. *Clinical Cancer Research*.

[6] Liedtke C, Mazouni C, Hess KR (2008). Response to neoadjuvant therapy and long-term survival in patients with triple-negative breast cancer. *Journal of Clinical Oncology*.

[7] Tanioka M, Shimizu C, Yonemori K (2010). Predictors of recurrence in breast cancer patients with a pathologic complete response after neoadjuvant chemotherapy. *British Journal of Cancer*.

[8] Pierga J-Y, Mouret E, Diéras V (2000). Prognostic value of persistent node involvement after neoadjuvant chemotherapy in patients with operable breast cancer. *British Journal of Cancer*.

[9] von Minckwitz G, Untch M, Blohmer JU (2012). Definition and impact of pathologic complete response on prognosis after neoadjuvant chemotherapy in various intrinsic breast cancer subtypes. *Journal of Clinical Oncology*.

[10] Farewell VT (1982). The use of mixture models for the analysis of survival data with long-term survivors. *Biometrics*.

[11] Othus M, Barlogie B, Leblanc ML, Crowley JJ (2012). Cure models as a useful statistical tool for analyzing survival. *Clinical Research Research*.

[12] Hirata T, Shimizu C, Yonemori K (2009). Change in the hormone receptor status following administration of neoadjuvant chemotherapy and its impact on the long-term outcome in patients with primary breast cancer. *British Journal of Cancer*.

[13] Allred DC, Harvey JM, Berardo M, Clark GM (1998). Prognostic and predictive factors in breast cancer by immunohistochemical analysis. *Modern Pathology*.

[14] Sy JP, Taylor JMG (2000). Estimation in a Cox proportional hazards cure model. *Biometrics*.

[15] Asano J, Hirakawa A, Hamada C (2013). A stepwise variable selection for a Cox proportional hazards cure model with application to breast cancer data. *Japanese Journal of Biometrics*.

